# An Incidental Ultrasonographic Diagnosis of Partial Hydatidiform Mole in a Old Primigravida: A Case Report

**DOI:** 10.31729/jnma.4575

**Published:** 2020-02-29

**Authors:** Sudeep Thapa, Ramesh Rana, Sheela Kumari

**Affiliations:** 1 Department of Radiology, Sidhababa Hospital and Research Centre Pvt. Ltd, Butwal, Nepal; 2 Department of Internal Medicine, Gautam Buddha Community Heart hospital, Butwal, Nepal; 3 Department of Obstetrics and Gynecology, Lumbini Provincial hospital, Butwal, Nepal

**Keywords:** *Hydatidiform mole*, *molar pregnancy*, *partial hydatidiform mole*

## Abstract

Hydatidiform mole is an abnormal pregnancy common in Asian populations compared to western countries; however, a partial hydatidiform mole is relatively uncommon and very challenging to diagnose on ultrasound. We reported a 24 years old female visited our clinic whose first antenatal visit was regular with normal viable fetus on ultrasonographic scan. However, an uneventful scan at 12 weeks period of gestation revealed a large irregular gestational sac with the disproportionately small embryo corresponding to 6^+6^ weeks with no cardiac activity. Additionally, there was markedly thickened placenta measuring 30mm in thickness without cystic spaces within the placenta. Further, her beta-human chorionic gonadotrophin level was very high and suction evacuation sample showed hydropic chorionic villi lined by trophoblastic cells with cistern formation and scalloped border. In conclusion, partial hydatidiform mole is an uncommon molar pregnancy rarely diagnose on ultrasonography. Late first-trimester scan and excessively high beta-human chorionic gonadotrophin levels confirm the diagnosis.

## INTRODUCTION

Hydatidiform mole (HM) is an abnormal pregnancy characterized by vesicular swelling of the chorionic villi, hyperplasia of the trophoblastic layer, and the absence or abnormal development of the fetus. It is the most common form of gestational trophoblastic disease. It is classified into a complete and partial hydatidiform mole. The incidence of HM range from 66-121/ 100,000 pregnancies in Europe and North America whereas much higher incidences in Asia and the Middle East.^1,2^ A triploid zygote leads to a Partial Hydatidiform Mole (PHM). Ultrasonographic diagnosis of molar pregnancy is very challenging, especially for partial mole. The average ultrasound detection rate for the complete mole is ∼80% whereas ∼30% for partial molar pregnancies.^3,4^ Recently magnetic resonance imaging has a significant role in evaluating the atypical presentation of hydatidiform mole.^5^

In this study, we reported an incidental diagnosis of partial molar pregnancy in 24 years old female primigravida at 6 weeks 6 days period of gestation by ultrasound.

## CASE REPORT

**Figure 1:**
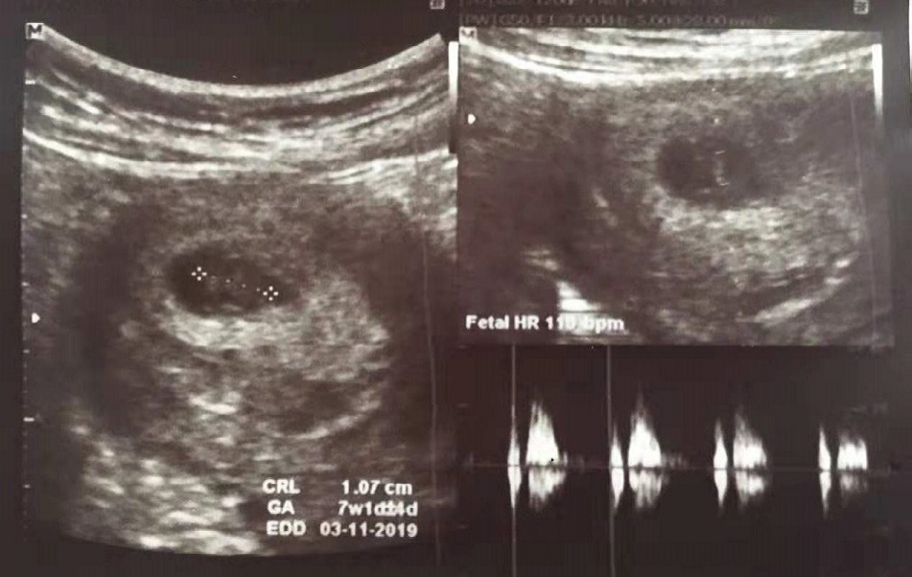
First antenatal visit USG scan showing viable fetus with fetal heart rate.

**Figure 2:**
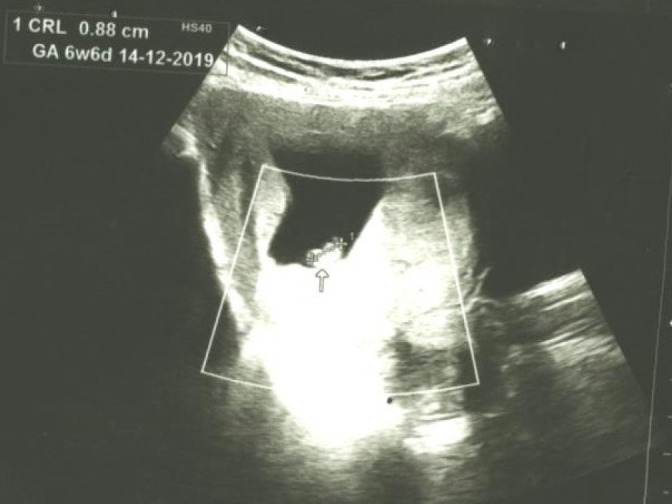
Ultrasonographic image of intrauterine fetal death without cardiac activity.

**Figure 3:**
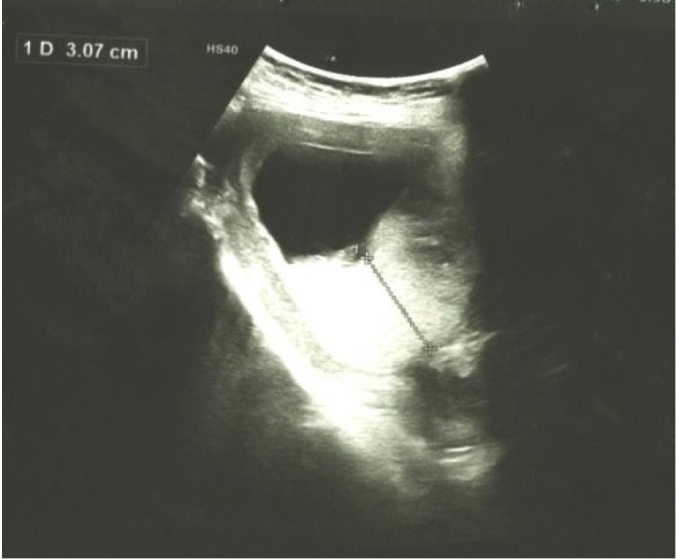
Ultrasonographic image of thickened placenta suggesting partial hydatidiform mole corresponding to age of gestation.

A 24 years old female, primigravida with a normal first antenatal visit at 7 weeks period of gestation (POG) presented with vomiting on and off in outpatient clinic. The transabdominal obstetric scan was done on first antenatal check-up elsewhere showed crown-rump length (CRL) 10.7mm (7 weeks 1 day) with a fetal heart rate of 110bpm (Figure 1). She denied of pain abdomen, per vaginal bleeding, or fever. Her vital parameters were stables. Transabdominal obstetric scan at 12 weeks POG was done for regular normal nuchal translucency measurement. It revealed a large irregular gestational sac with the disproportionately small embryo (CRL 8.8 mm, corresponding to 6 weeks 6 days) with no cardiac activity. Additionally, there was markedly thickened placenta measuring of 30mm in thickness. However, no cystic spaces noted within the placenta (Figure 2 and 3). The provisional diagnosis was made of gestational trophoblastic disease with suspected partial mole and serum beta-human chorionic gonadotropin (B-hCG) level was investigated. The lab investigation report showed remarkable raised B-hCG level of 76,670 mIU/ ml. Other routine investigations were within normal limit including normal thyroid function test. Subsequently, the suction evacuation was performed and the sample was sent for histopathology. She was kept on regular follow up with serial (B-hCG) level which revealed progressively declined on post suction evacuation days; 3 days (14,480 mIU/ml), 10 days (1880 mIU/ ml), 22 days (84 mIU/ml), 37 days (17.1 mIU/ml), and normal in 2 months. Furthermore, histopathologically, it demonstrated multiple pieces of grey-brown tissue and cut section showing the grape-like structure on gross examination and microscopic section showed hydropic chorionic villi lined by trophoblastic cells with cistern formation and scalloped border which is suggestive of partial hydatidiform mole. She is under a regular follow up with B-hCG monitoring.

## DISCUSSION

The gestational trophoblastic disease covers a spectrum of benign and malignant conditions occurred due to malformed pregnancies. Complete and partial hydatidiform moles are benign form, however, these increase the risk of further molar pregnancies (1-2%) and even malignant transformation.^6^ The incidence of hydatidiform mole is higher in Asia around 1 in 500 and the Middle East and Africa ∼1 in 1000 compared to Europe and North America of ∼1 in 1500.^2,7^

Partial hydatidiform mole is a triploid androgenetic pregnancy having an enlarged placenta and abnormal fetus. It is more difficult to diagnose in early pregnancy as it mimics a normal pregnancy and ultrasonographic diagnosis of PHM is ∼30% only.^3^ Therefore, PHM is suspected in the late first-trimester when there is the visibly thickened placenta. Moreover, intrauterine fetal growth retardation, structural abnormalities, and placental cystic changes are observed on ultrasound during the second trimester (Table 1). B-hCG levels establish the diagnosis.^5^ In our case, her first antenatal visit was normal with normal intrauterine early pregnancy and fetal heart rate was present on the obstetric scan. However, a late first-trimester uneventful obstetric scan showed notable thickened placenta and intrauterine fetal demise suggestive of molar pregnancy (partial hydatidiform mole) which was later confirmed by excessively high serum B-hCG levels and microscopically hydropic chorionic villi lined by trophoblastic cells with cistern formation and scalloped border which is suggestive of PHM.^8,9^ There is an increased risk of malignant transformation in high-risk hydatidiform mole with the incidence of post mole neoplasia around 8% despite good prognosis with chemotherapy.^10^

In conclusion, although hydatidiform moles are more common in Asian populations, however, partial hydatidiform moles are relatively uncommon and difficult to diagnose with ultrasonography. The late first-trimester obstetric scan increases the accuracy of diagnosing molar pregnancy.

**Consent:** JNMA Case Report Consent Form was signed by the patient and the original article is attached with the patient’s chart.

## Conflicts of Interest:

None.
